# Surgical results regarding the correction of macular hole with and without face-down posturing using 25% SF6 gas: a retrospective case series

**DOI:** 10.5935/0004-2749.20200044

**Published:** 2024-02-11

**Authors:** Andre Luis Carvalho de Moura Bastos, Marcos Pereira de Ávila, David Leonardo Cruvinel Isaac, Levy Paz Aguiar, Aline Guerreiro Aguiar

**Affiliations:** 1 Department of Ophthalmology, Humberto Castro Lima Hospital, Salvador, BA, Brasil; 2 Department of Ophthalmology, Centro Brasileiro de Cirurgia de Olhos, Goiânia, GO, Brasil

**Keywords:** Retinal perforations, Vitrectomy, Vitreoretinal surgery, Sulfur hexafluoride/administration & dosage, Fluorocarbons/ administration & dosage, Supine position, Postoperative care, Perfurações retinianas, Vitrectomia, Cirurgia vitreorret iniana, Hexafluoreto de enxofre/administração & dosagem, Fluorocarbonetos/administração & dosagem, Decúbito dorsal, Cuidados pós-operatórios

## Abstract

**Purpose:**

This study aims to compare the anatomical success rates of vitrectomy and SF6 gas tamponade for macular hole surgery with and without postoperative face-down posturing.

**Methods:**

This was an observational, longitudinal, and retrospective case series analysis. The study included 52 eyes from 52 patients who underwent pars plana vitrectomy with trypan blue-assisted internal limiting membrane peeling and 25% SF6 tamponade for stages 2, 3, and 4 macular holes. After surgery, all patients were provided with a postoperative postural regimen: 31 patients were instructed not to maintain face-down posturing, whereas 21 were instructed to maintain face-down posturing for 7 days. The primary outcome measure was the macular hole closure rate. Statistical analysis was performed using Epi Info 7.1.

**Results:**

A total of 47 (90.3%) patients achieved hole closure. The nonface-down posturing group and face-down posturing group obtained closure rates of 90.3% and 90.4%, respectively; these rates were not significantly different. Statistical analysis revealed that no significant differences existed in sex, age, hole duration, hole stage, preoperative visual acuity, or postoperative visual acuity between the two groups.

**Conclusion:**

Our results suggest that macular hole surgery with the use of short duration gas (SF6) is safe and effective and that maintaining a postural orientation of nonface-down posturing is also safe. However, these recommendations should be assessed further in a prospective and randomized study to comprehensively delineate the associated benefits and risks.

## Abstract (pt)

**Objetivos:**

Comparar as taxas de sucesso anatômico da vitrectomia e tamponamento de gás SF6 na cirurgia de buraco macular com e sem a postura pronada pós-operatória.

**Métodos:**

Foi realizado um estudo observacional, longitudinal e retrospectivo de séries de casos. O estudo incluiu 52 olhos de 52 pacientes submetidos à vitrectomia posterior via pars-plana com *peeling* de membrana limitante interna auxiliada por azul *trypan* e tamponamento com gás SF6 a 25% para os estágios 2, 3 e 4 dos buracos maculares. Após a cirurgia, todos os pacientes foram orientados a manter um regime postural pós-operatório: 31 pacientes foram orientados a não realizar posição pronada de cabeça, enquanto 21 foram orientados a manter uma pronada pós-operatória por 7 dias. O objetivo principal foi a análise da taxa de fechamento do buraco macular. A análise estatística foi realizada usando Epi-Info 7.1.

**Resultados:**

Um total de 47 (90,3%) pacientes obtiveram fechamento do buraco macular. O grupo de postura não pronada e o grupo de postura pronada obtiveram taxas de fechamento de 90,3%, e 90,4%, respectivamente; essas taxas não foram significativamente diferentes. A análise estatística revelou que não houve diferenças significativas relacionadas ao gênero, idade, duração do buraco macular, estágio do buraco macular, acuidade visual corrigida pré e pós-operatória entre os dois grupos.

**Conclusão:**

Nossos resultados sugerem que a cirurgia para buraco macular com o uso de gás de curta duração (SF6) é segura e eficaz e que a manutenção de uma orientação pós-operatória de não-pronada também é segura. No entanto, essas recomendações devem ser avaliadas em um estudo prospectivo e randomizado para delinear de forma abrangente os riscos e benefícios associados.

## INTRODUCTION

Most macular holes (MHs) are idiopathic and occur in patients with no history of ocular diseases between the sixth and seventh decades of life. Although the pathophysiology of MH is not fully known, MH formation is strongly associated with factors such as the presence of anteroposterior and tangential traction at the level of the vitreoretinal interface^(1)^.

More recently, the advent of optical coherence tomography (OCT) has facilitated the assessment of MH formation. Sequential OCT scanning suggested that vitreofoveal anteroposterior traction may constitute an underlying mechanism in MH development, thus corroborating the theories of hole pathogenesis. This mechanism provides good justification for the use of vitrectomy in the treatment of MHs^(1-3)^.

Corrective surgery for MHs has been performed for more than 15 years with few changes in technique since the initial description; it consists of pars plana vitrectomy, posterior vitreous removal, vitreous cavity filling with a gas bubble, and postural orientation treatment for 1 week. This type of surgery aims to relieve the traction forces and stimulate the healing processes of MH^(4)^.

Some authors prefer to use long-acting gas (C3F8) in MH surgeries because they presume that longer tamponade time is associated with higher rates of anatomical success^(5)^. However, OCT studies showed MH closure on the first postoperative day^(6-8)^. Recent studies have shown that there are no statistically significant differences in anatomical or visual success between C3F8 and SF6 gases^(9-14)^.

The need for face-down posturing (FDP) remains open to discussion and controversy because eliminating the need for FDP would allow the surgery to be extended to a greater number of patients, particularly those with postural or psychological limitations, with greater postoperative comfort and reduced need for a postoperative rest period^(15)^. Thus, this study was performed to analyze the results of MH surgery using short duration gas (SF6) and the differences in anatomical success rate between patients instructed to maintain FDP orientation and those who were only instructed to avoid the supine position.

## METHODS

This observational, longitudinal, retrospective case series analysis was conducted at the Instituto Brasileiro de Oftalmologia e Prevenção da Cegueira to evaluate the anatomical success rate of surgery for idiopathic MH correction. This study included patients >50 years old who were diagnosed with idiopathic MH in the II, III, or IV stages of Gass, as confirmed by indirect binocular ophthalmoscopy and OCT (Stratus OCT^®^, Carl-Zeiss, San Leandro, CAUSE). There were no size limits to MHs in the study; they ranged from <250 µm to >400 µm. Patients were excluded from this study if they were younger than 50 years of age; if they had systemic disease that prevented surgery; if they had diabetic retinopathy, MH in a myopic eye, or secondary MH (secondary to trauma, uveitis, or cystic macular edema); and if they had previously undergone posterior vitrectomy surgery.

After diagnostic confirmation, all patients underwent complete ophthalmologic examination, which consisted of the measurement of best corrected visual acuity (BCVA, Snellen ratios), anterior and posterior segment biomicroscopy, retinal examination using indirect ophthalmoscopy of 20 diopters with scleral depression, applanation tonometry with a Goldmann tonometer, and ocular ultrasonography with Ultrascan Alcon. All patients were examined by the investigator. OCT examination was performed using a technique certified by the manufacturer.

All surgeries were performed by the same surgeon (ALCMB) and consisted of posterior vitrectomy with hyaloidectomy and internal limiting membrane (ILM) peeling by dyeing with 0.05% Brilliant Blue (OphtBlue, R Ophtalmos, Brazil) and Eckardt 23 gauge forceps (Synergetics, USA). Vitrectomy was performed using a 23-gauge technique with the Accurus system (Alcon, USA) and was visualized using a wide-angle contact lens (Volk, Miniquad, USA) and Machemer macula (Ocular, USA) or Woldoff NA High Magnification (Ocular, USA).

After ILM peeling and peripheral retina examination, a complete fluid air exchange was performed and 25% SF6 gas was injected. The sealing of incisions was evaluated. In patients with leakage, the incision was sutured with a 7-0 polyglactin multifilament thread. After surgery, all patients were given instructions regarding a postoperative postural regimen. The patients were divided into two groups on the basis of this orientation:

Group I (non-FDP): Patients were instructed not to maintain FDP. Patients were free to walk and sit normally, as well as to lie in lateral and ventral decubitus positions. They were instructed to avoid lying in the supine position at any time during the first seven postoperative days.

Group II (FDP): Patients were instructed to maintain FDP as long as possible during the first seven postoperative days and were unable to lie in lateral and dorsal decubitus positions. The two groups were defined via the retrospective analysis of medical records, and there were no previously defined criteria for the inclusion of patients in any of the groups.

All patients in both groups were advised to use Predfort^®^ (1.0% prednisolone acetate, Allergan, BR) for 20 days, Zymar^®^ (0.3% gatifloxacin, Allergan) for 7 days, and Accular LS^®^ (0.4% tromethamine ketorolac, Allergan) for 30 days, in addition to avoiding lying in the supine position during the first postoperative week.

The patients were evaluated on the 1st, 7th, 30th, and 90th postoperative days. The anatomical success rate of surgery (closure of the MH) was determined by OCT at the end of the first postoperative month. In cases of surgical failure ( nonclosure of the MH) found after OCT in group I patients at the end of the first postoperative month, 25% SF6 gas was reinjected, and the patient was instructed to maintain FDP for seven days. BCVA was measured before surgery and on the 90th postoperative day by Snellen assessment. Visual success was regarded an improvement of two or more lines in Snellen acuity assessment.

Statistical analysis was performed using Epi Info 7.1 for Windows. Qualitative variables were described using simple and relative frequency tables. For comparisons between the FDP and non-FDP groups regarding the events of interest, the chi-square test or Fisher’s exact test was used. A p-value <0.05 was considered statistically significant.

## RESULTS

Among the 52 patients evaluated, 31 patients (60%) and 21 patients (40%) were in the non-FDP group and FDP group, respectively. The mean age of the patients was 64 years (range: 53-82 years). Regarding sex, 44 patients (85%) were women, and 8 patients (15%) were men. The mean duration of MH presence was 17 months, with a maximum of 36 months. Among the 52 patients, 21 patients (40.5%) had MH for ≤12 months, 21 patients (40.5%) had MH for 13-24 months, and 10 patients (19%) had MH for >24 months. Regarding the Gass stages of MHs, 2 patients (4%) had stage II, 14 patients (27%) had stage III, and 36 patients (69%) had stage IV. Table 1 shows a summary of the demographic and clinical characteristics; no statistically significant differences were found between groups.

**Table 1:** Clinical and demographic characteristics of patients

	Non-FDP group n (%)	FDP group n (%)	P-value^*^
Sex			
Male	6(20)	2(10)	0.3277
Female	25 (80)	19(90)	
Age, years			
Mean ± SD	63.67 ± 6.9	64.09 ± 5.0	0.6202
Range	(54-82)	(53-72)	
Time of MH, months			
Mean ± SD	17.00 ± 9.1	17.42 ± 7.7	0.7792
Range	(6-36)	(6-30)	
Grade hole (Gass)			
11	1 (3.2)	1 (4.8)	0.5670
111	10(32.3)	4(19)	
IV	20 (64.5)	16 (76.2)	

* A chi-square test was used; MH= macular hole; SD= standard deviation; FDP= face-down posturing.

**Figure 1:**
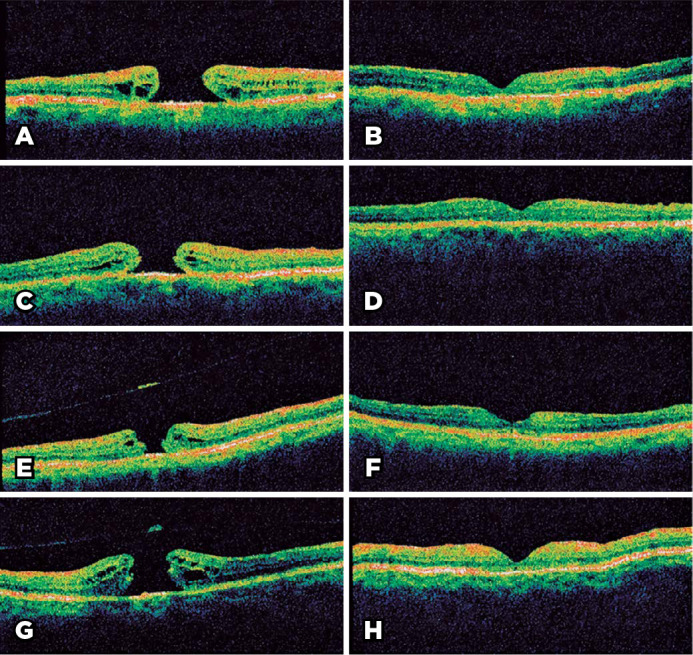
Spectral domain OCT images of patients before surgery (A, C, E, G) and 30 days after surgery (B, D, F, H). Representative patients: a 66-year-old woman from the non-FDP group (A and B) with an initial BCVA of 20/200 and postoperative BCVA of 20/100, a 71-year-old woman from the non-FDP group (C and D) with an initial BCVA of 20/400 and postoperative BCVA of 20/80, a 62-year-old woman from the FDP group (E and F) with an initial BCVA of 20/200 and postoperative BCVA of 20/60, and a 62-year-old woman from the FDP group (G and H) with an initial BCVA of 20/400 and postoperative BCVA of 20/80.

Baseline BCVA was worse than or equal to 20/200 in 38 patients (73%). After surgical treatment, 27 patients (52%) exhibited BCVA equal to or better than 20/60; 36 patients (69.23%) had visual improvement of two or more lines in Snellen acuity assessment. The MH closure rate was 90.3% (47/52 patients). Twenty-eight of 31 patients (90.3%) in the non-FDP group achieved anatomical success, similar to 19/21 patients (90.4%) in the FDP group (Figure 1); this difference was not statistically significant (P=0.6803). Five patients did not achieve anatomical success and underwent reinjection of SF6 gas; they were instructed to maintain FPD for 7 days, but none achieved MH closure. Among the patients in the non-FDP group, 23 patients (74.19%) had visual improvement of two or more lines in Snellen acuity assessment (i.e., they achieved visual success), whereas 13 patients (61.90%) in the FDP group had the same outcome; the results were not significantly different (P=0.2613). Table 2 presents a summary of these results.

**Table 2:** Postoperative outcomes

	Non-FDP group n (%)	FDP group n (%)	p-value^*^
Anatomical success Yes No	28 (90.3) 3 (9.7)	19 (90.5) 2 (9.5)	0.6803
Visual success Yes No	23 (74.2) 8 (25.8)	13 (61.9) 8(38.1)	0.2613

* Fisher’s exact test was used; FDP= face-down posturing; Visual success= improvement of two or more lines in Snellen acuity assessment.

## DISCUSSION

Since the introduction of MH surgery, postoperative FDP has been routinely performed. One theory regarding the importance of FDP states that the gas bubble on the macula isolates the MH area from the vitreous fluid, thereby maintaining a dry macula^(5,16-18)^. Many studies have shown results that lead to questions regarding the need for FDP^(8,15-22)^. OCT studies have shown MH closure on the first postoperative day^(6-8)^.

In this study, 28/31 of the patients (90.3%) in the non-FDP group had anatomical success, and 23/31 of the patients (74.19%) had visual success; these results were not significantly different from those of patients in the FDP group, in which 19/21 (90.4%) and 13/21 (61.90%) had anatomical success and visual success, respectively. The results achieved in the non-FDP group are similar to those of other studies in which patients-maintained FDP and studies in which patients did not maintain FDP. A systematic review in 2009 examined five studies in which patients underwent MH surgery without the main tenance of FDP; the MH closure rate ranged from 87.5%-92%, but all studies used long-term gas (four studies used C3F8 gas and one study used C2F6 gas)^(23,24)^.

In our study, the duration of MH presence was a predictor of anatomical and visual success: MHs present for ≤12 months had a 100% closure rate, MHs present for 13-24 months had a 95% closure rate, and MHs present for >24 months had a 60% closure rate (P=0.0012). The visual success rate in patients with MHs present for ≤12 months was 85.7%, whereas the visual success rate in patients with MHs present for >24 months was 20% (P=0.0007).

Despite the limitations of our study, namely, short postoperative follow-up time and limited number of patients, our results and those of prior studies suggest that the use of long-term gas is not routinely required for MH surgery. Moreover, the postoperative maintenance of FDP is not required. These measures may be reserved for patients with recurrent MHs or those at high risk of recurrence. However, a large prospective case series or randomized clinical trial is needed (e.g., a comparison of postoperative regimens with and without the maintenance of FDP after MH correction surgeries using short duration gas) to fully delineate the efficacy and complications of the present recommendations.

## References

[1] Gass JD. (1988). Idiopathic senile macular holes: its early stages and
pathogenesis. Arch Ophthalmol.

[2] Chan A, Duker JS, Schuman JS, Fugimoto JG. (2004). Stage 0 macular holes: observations by optical coherence
tomography. Ophthalmology.

[3] Avila MP, Jalkh AE, Murakami K, Trempe CL, Schepens CL. (1983). Biomicroscopic study of the vitreous in macular
breaks. Ophthalmology.

[4] Kelly NE, Wendel RT. (1991). Vitreous surgery for idiopathic macular holes. Arch Ophthalmol.

[5] Thompson JT, Smiddy WE, Glaser BM, Sjaarda RN, Flynn HW (1996). Intraocular tamponade duration and success of macular hole
surgery. Retina.

[6] Michel JJ, Gallemore RP, McCuen BW, Toth CA. (2000). Features of macular hole closure in the early post-operative
period using optical coherence tomography. Retina.

[7] Satchi K, Patel CK. (2005). Posterior chamber compartments demonstrated by optical coherence
tomography, in silicone filled eyes, following macular hole
surgery. Clin Exp Ophthalmol.

[8] Eckardt C, Eckert T, Eckardt U, Porkert U, Gesser C. (2008). Macular hole surgery with air tamponade and optical coherence
tomography-based duration of face-down positioning. Retina.

[9] Essex RW, Kingston ZS, Moreno-Betancur M, Shadbolt B, Hunyor AP, Campbell WG, Connell PP, McAllister IL, Australian and New Zealand Society of Retinal Specialists Macular
Hole Study Group (2016). The effect of postoperative face-down positioning and of
longversus short-acting gas in macular hole surgery: results of a
registry-based study. Ophthalmology.

[10] Kim SS, Smiddy WE, Feuer WJ, Shi W. (2008). Outcomes of sulfur hexafluoride (SF6) versus perfluoropropane
(C3F8) gas tamponade for macular hole surgery. Retina.

[11] Briand S, Chalifoux E, Tourville E, Bourgault S, Caissie M, Tardif Y (2015). Prospective randomized trial: outcomes of SF6 versus C3F8 in
macular hole surgery. Can J Ophthalmol.

[12] Modi A, Giridhar A, Gopalakrishnan M. (2017). Sulfurhexafluoride (sf6) versus perfluoropropane (c3f8) gas as
tamponade in macular hole surgery. Retina.

[13] Casini G, Loiudice P, De Cillà S, Radice P, Nardi M. (2016). Sulfur hexafluoride (SF6) versus perfluoropropane (C3F8)
tamponade and short-term face-down position for macular hole repair: a
randomized prospective study. Int J Retina Vitreous.

[14] Xirou T, Theodossiadis PG, Apostolopoulos M, Kabanarou SA, Feretis E, Ladas ID (2012). Macular hole surgery with short-acting gas and short-duration
face-down positioning. Clin Ophthalmol.

[15] Merkur AB, Tuli R. (2007). Macular hole repair with limited nonsupine
posturing. Retina.

[16] Tranos PG, Peter NM, Nath R, Singh M, Dimitrakos S, Charteris D (2007). Macular hole surgery without prone posturing. Eye (Lond).

[17] Krohn J. (2005). Duration of face-down positioning after macular hole surgery: a
comparison between 1 week and 3 days. Acta Ophthalmol Scand.

[18] Krohn J. (2003). Topical medication interferes with face-down positioning after
macular hole surgery. Acta Ophthalmol Scand.

[19] Nadal J, Delas B, Piñero A. (2012). Vitrectomy without face-down posturing for idiopathic macular
holes. Retina.

[20] Dhawahir-Scala FE, Maino A, Saha K, Mokashi AA, McLauchlan R, Charles S. (2008). To posture or not to posture after macular hole
surgery. Retina.

[21] Simcock PR, Scalia S. (2001). Phacovitrectomy without prone posture for full thickness macular
holes. Br J Ophthalmol.

[22] Rubenstein A, Ang A, Patel CK. (2007). Vitrectomy without postoperative posturing for idiopathic macular
holes. Clin Exp Ophthalmol.

[23] Johnson MW. (2002). Improvements in the understanding and treatment of macular
hole. Curr Opin Ophthalmol.

[24] Gupta D. (2009). Face-down posturing after macular hole surgery: a
review. Retina.

